# A case report of primary intrahepatic adeno squamous cell cholangiocarcinoma

**DOI:** 10.1016/j.ijscr.2024.110366

**Published:** 2024-09-30

**Authors:** Fionn Woulfe, Michael Devine, Brian Hayes, Rory Crotty, Adrian O'Sullivan

**Affiliations:** a Mercy University Hospital, Grenville Place, Cork T12 WE28, Ireland; b Hermitage Blackrock Clinic/Royal College of Surgeons of Ireland, 121 St.Stephen's Green, Dublin, Ireland; c Cork University Hospital, Wilton Road, Wilton, Cork T12 DC4A, Ireland

**Keywords:** Hepatocellular carcinoma, Primary keratinising squamous cell carcinoma, Adeno squamous carcinoma, Intrahepatic cholangiocarcinoma, Liver abscess

## Abstract

**Introduction:**

Hepatocellular carcinoma is the most common form of primary liver cancer. Intrahepatic cholangiocarcinoma and fibrolamellar carcinoma make up most other cases. The vast majority of intrahepatic cholangiocarcinoma's are adenocarcinoma in nature. Few reports have indicated pure squamous cell or mixed squamous glandular histopathology.

**Presentation of case:**

We present the case of a 35-year-old female whose preoperative diagnosis indicated primary keratinizing squamous cell carcinoma (SCC) of the liver. However, histological analysis of surgical resections later confirmed intrahepatic cholangiocarcinoma composed of 95 % squamous and 5 % glandular features.

**Discussion:**

The change in diagnosis post-operatively is indicative of the pre-operative diagnostic difficulties associated with these newly classified variants. While adenomatous differentiation is the most common form of intrahepatic cholangiocarcinoma, a squamous and mixed histology can be observed.

**Conclusion:**

Surgeons must be aware of new histological variants of cholangiocarcinoma, potential differentials, and direct further research to improve their poor prognosis.

## Highlights

• Rare variants of intrahepatic cholangiocarcinoma exist.
• Intrahepatic cholangiocarcinoma can present with a predominantly squamous histopathological differentiation.
• Biopsy proven primary SCC of the liver may be a misrepresentation of intrahepatic cholangiocarcinoma.

## 1 Introduction

Liver cancer is the 6th most common cancer worldwide [1]. Hepatocellular carcinoma (HCC) represents approximately 80 % of primary liver cancers. Intrahepatic cholangiocarcinoma (ICC) and a mixed HCC-ICC form most other cases [2]. Cholangiocarcinoma can be characterized anatomically based upon its location: intrahepatic, perihilar and distal. Intrahepatic cholangiocarcinoma makes up approximately 10 % of all cholangiocarcinoma's [3].

Intrahepatic cholangiocarcinoma can be divided into three histological growth types based on appearances: mass forming, periductal, and intraductal infiltrating [3]. In contrast with HCCs, ICCs are white and firm as they contain more desmoplastic stroma. The formation of ICC is often due to mutations in the KRAS oncogene, and the deletion of the p53 gene [3].

Traditionally intrahepatic cholangiocarcinoma's were considered adenocarcinomas. However, new classifications by the world health organization acknowledges exceedingly rare variants including adenosquamous, squamous and mucinous types [4]. We report a case in line with the SCARE criteria, which details the challenges of diagnosing and treating these rare entities [5].

## 2 Presentation of case

A female in her 30's presented to the emergency department of a tertiary hospital with a four-month history of right upper quadrant pain, night sweats and fever. She reported unintentional weight loss over the preceding months. Nausea, vomiting, anorexia, hypodynamia or bowel changes were not present. Her co-morbidities included migraine and recurrent idiopathic pancreatitis. There was no history of hepatic infection or other liver insult. Our patient was a non-smoker who consumed alcohol rarely, and in moderation. On physical examination she had a normal Body Mass Index. Tenderness was elicited in the right upper quadrant and a palpable mass extended beneath the right subchondral border. Systemic examination revealed no other stigmata of illness, or specifically of liver disease. Bloods included Hb: 12.3 (normal range 12–16), WBC: 13.8 (normal range 4.5–11), Neutrophils: 10.6 (normal range 2.5–7), CRP 83.1 (normal range 0–10), and B-HCG: 156.5 (normal range < 5). Liver function markers were unremarkable. Other tumour markers were CA19–9: 556 U/ml (normal <37), Alpha-fetoprotein 1.1 (normal <8.8), and CEA: 1.7 (normal <5).

Ultrasound of the liver revealed an irregular bilobed cystic lesion in the hepatic subcapsular space measuring 6 cm. Dependent debris/haemorrhage was noted within the cystic lesion with no internal flow or hyperaemia of the surrounding hepatic parenchyma. There was no clear communication with the biliary tree.

On MRI, a large mixed solid cystic lesion measuring 5.1 × 3.2 × 2.1 cm was identified in the right lobe of the liver. Restricted diffusion and areas of nodular enhancement were present. These appearances favoured a primary hepatic tumour over a hepatic abscess. CT indicated a multilocular cystic and low-density lesion in the right lobe of the liver with secondary ductal dilatation and involvement of the liver capsule.

**Fig. 1:**
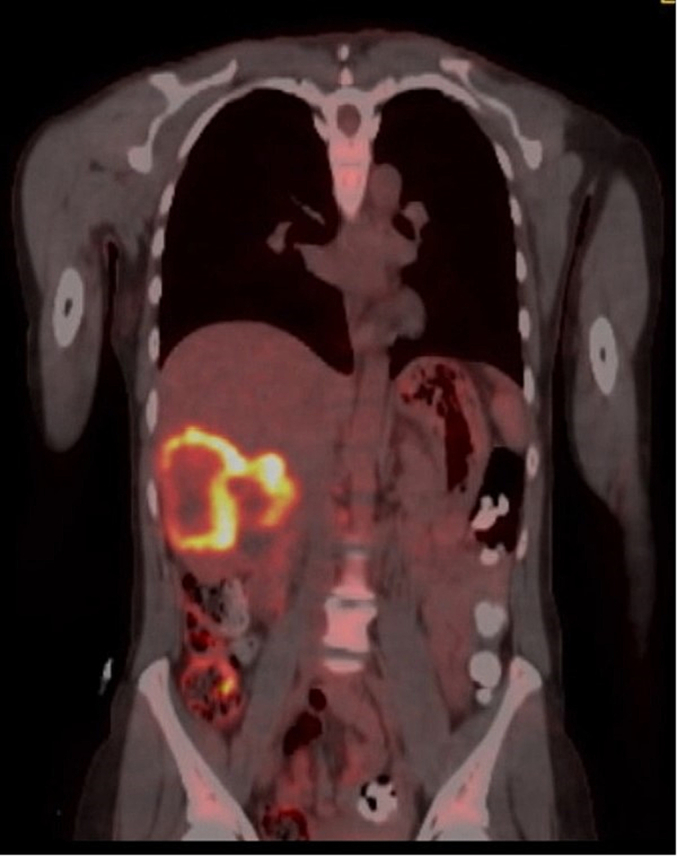
PET scan – coronal view.

Ultrasound guided liver biopsy demonstrated moderately differentiated squamous cell carcinoma of the liver with focal keratinization. A PET scan [Fig. 1. PET Scan – Coronal View], OGD and gynaecological review were arranged to rule out primary disease of the uterus and oesophagus. Negative findings further supported the diagnoses of a primary hepatic malignancy.

**Fig. 2:**
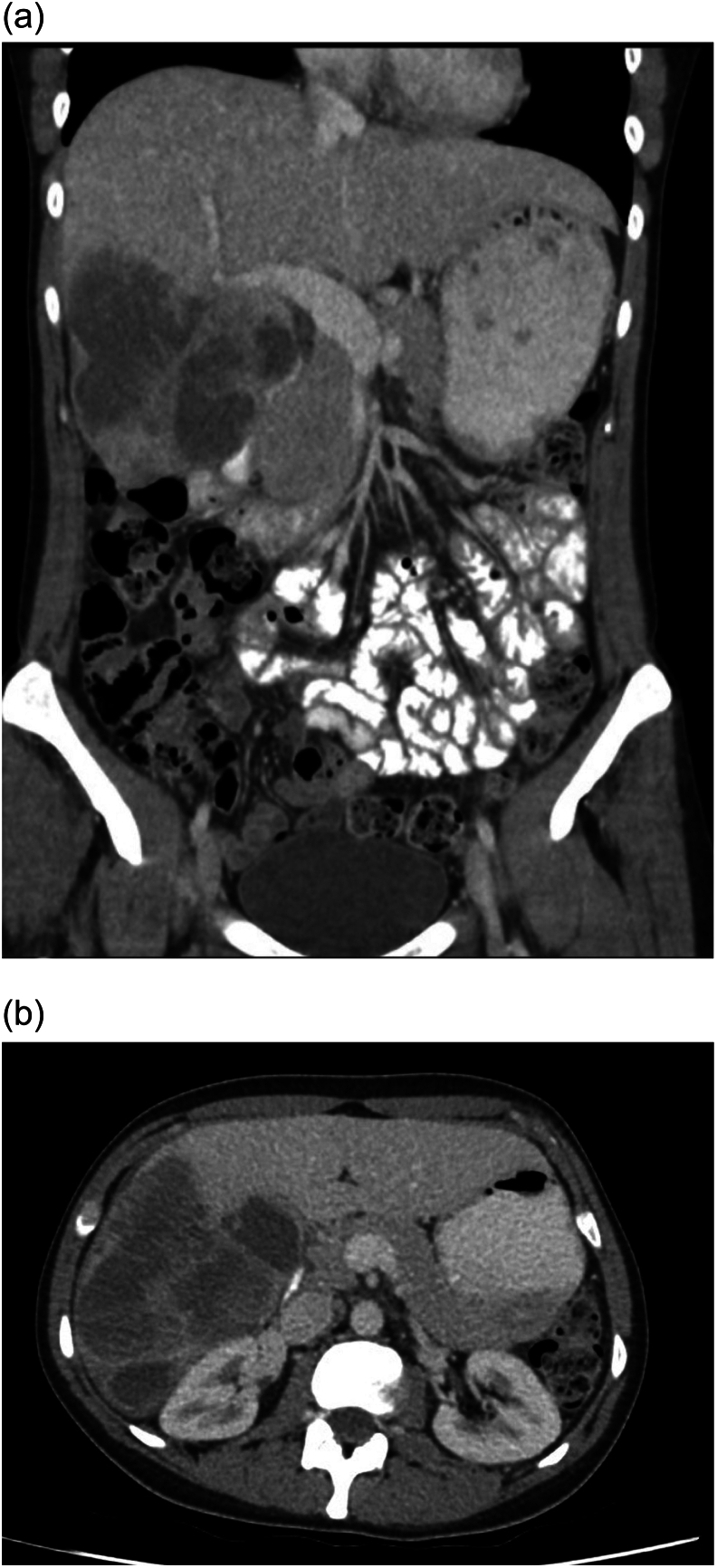
CT liver - coronal & axial view.

**Fig. 3:**
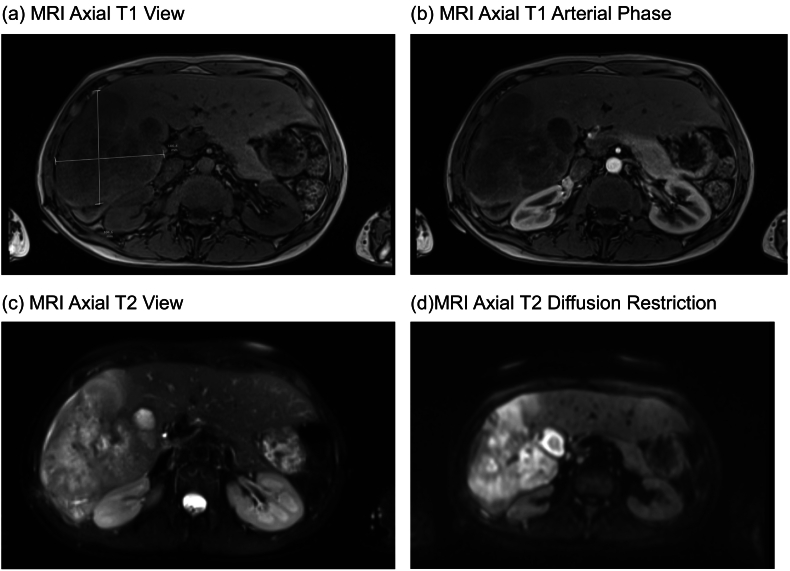
MRI liver – axial T1 view, T1 arterial phase, T2 View, & T2 diffusion restriction.

A repeat CT Abdomen Pelvis was performed one month after initial imaging which demonstrated multiple masses in the right lobe of the liver showing an increase in size since the previous CT scan [Fig. 2. CT Liver - Coronal & Axial View]. MRI liver was also repeated showing a large mass lesion replacing most of hepatic segments V and VI, extending into VII and VIII. It measured 10 cm in the transverse diameter, 13 cm in height and 10 cm in anteroposterior depth [Fig. 3. MRI Liver – Axial T1 View, T1 Arterial Phase, T2 View, & T2 Diffusion Restriction].

Consideration of clinical stigmata, biochemical results, histopathological features, and serial radiological images, lead the multidisciplinary team to favour a consensus of primary keratinizing SCC.

**Fig. 4:**
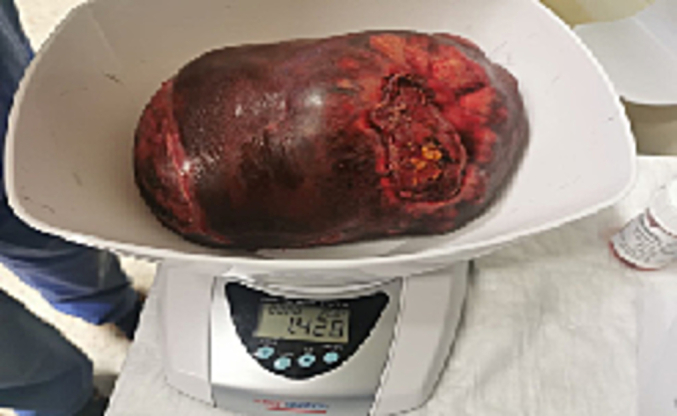
Intra-operative resection.

Definitive surgery was indicated given the rapid progression of disease and increasing symptoms. Following the hepatobiliary multidisciplinary meeting a right hemi hepatectomy, cholecystectomy and lymphadenectomy was performed by a hepatobiliary attending. The resected liver specimen weighed 1.428 kg [Fig. 4. Intra-operative Resection]. Operative time was four hours and thirty minutes. Estimated blood loss was 1 l. No intra-operative complications occurred. The patient had an ileus post operatively, and subsequently her bowels opened on day six. Her post-operative hospital stay was twelve days, after which she was discharged home.

**Fig. 5:**
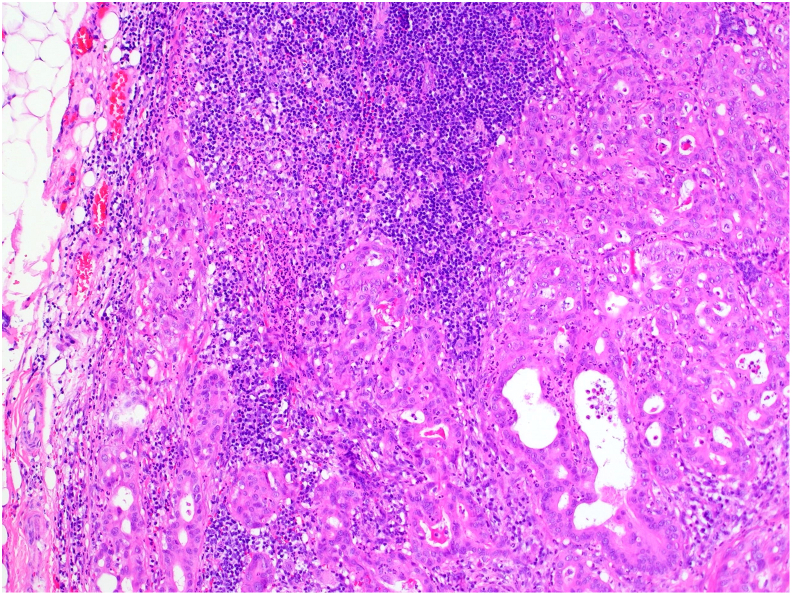
Glands in node at ×40 magnification.

Histological examination of resected specimens revealed extensive squamous differentiation with small, scattered foci of well differentiated glandular tissue (95 % Squamous, 5 % glandular features). The tumour growth pattern was mass forming. There was no evidence of cirrhosis, or of any specific hepatitis or cholangiopathic disorder in the background liver. Resected lymph nodes were composed of essentially exclusive glandular tissue (Fig. 5. Glands in Node at 40 Magnification) (metastatic carcinoma identified in 2/2 retro pancreatic lymph nodes, 2/4 aorto-caval lymph nodes and 0/6 common hepatic lymph nodes).

Following histopathological review, the final diagnosis of a primary cholangiocarcinoma of the liver with 95 % squamous differentiation was agreed.

The patient was discharged from hospital and received adjunctive Gemcitabine and Cisplatin as agreed by MDT consensus. Based on MSI and Oncomine sequencing assays Dervalumab was administered. Unfortunately, the patient died five months post operatively, seven months following initial diagnosis secondary to disease progression with metastasis.

## 3 Discussion

Cholangiocarcinoma, hepatocellular carcinoma, liver metastasis and liver abscess represent potential differentials which ought to be considered in this clinical case. Clinical presentations of these diseases however are vague, non-specific and overlap.

Our patient presented with abdominal discomfort, weight loss, fever, and malaise. Other features of liver disease may include jaundice, pruritus, anorexia, anasarca, diarrhoea, variceal bleeding, ascites, and progressive dysphagia [[5], [6], [7]]. While steatorrhea and dark urine may occur in cholangiocarcinoma, they're more common in those with perihilar or distal, rather than intrahepatic disease [8]. Fever on initial presentation of HCC and ICC is uncommon, and associated with a poorer prognosis [9].

While specific risk factors exist for ICC, HCC, and liver abscesses, many risk factors are associated to all. Liver cirrhosis, viral infections, increasing age, male sex, alcoholism, and diabetes mellitus are examples [10,11]. Specific risk factors to be considered for intrahepatic cholangiocarcinoma include primary sclerosing cholangitis, choledocholithiasis, choledochal cysts, liver fluke infection, inflammatory bowel disease, and anatomical variations in the bile and pancreatic ducts [11]. No specific risk factors for adenosquamous ICC have been identified [12].

Interestingly our patient was a young female with no underlying medical conditions. Her social history and demographics placed her at low risk. Other limited reports of ICC with squamous differentiation have also stated liver cirrhosis was not present [4]. Our patient had two hepatic cysts present, however. While the prevalence of liver cysts in the general population is not uncommon, most are benign entities [10,13]. That said, transformation to malignant disease has been documented in the literature with a small subset representing neoplastic precursors to cholangiocarcinomas [13].

Pre surgical biopsies of the lesion indicated a primary squamous cell carcinoma of the liver. Primary squamous cell carcinoma is a rare form of primary liver cancer with merely 32 documented cases in the literature to date [16,17]. At surgery, hilar lymph nodes were negative for disease, however the retro pancreatic and aortocaval nodes were prominent. Their histological composition indicated an essentially exclusive glandular composition. In contrast, the intrahepatic tumour demonstrated an extensive squamous composition. Overall, the histological and immunohistochemical findings were most consistent with intrahepatic cholangiocarcinoma with extensive squamous differentiation within the liver and glandular differentiation in the lymph nodes. Like primary squamous cell carcinoma, intrahepatic adenosquamous cholangiocarcinoma (ASC) also carries a poor prognosis with few being reported in the literature [12,15]. The positive expression of CK 19 confirmed the bile ductular ontogeny of the neoplastic cells, commonly expressed in intrahepatic cholangiocarcinoma [14]. Indeed, the histopathological composition of the tumour differs from most ICCs with glandular/adeno composition typically being most common [15].

There are no specific features or signatures of primary hepatic adenosquamous cell cholangiocarcinoma on CT or ultrasonography [12]. This exacerbates the difficulty in defining diagnostic criteria for primary hepatic ASC. In our case, the patients admission bloods were, WBC 13.8, Neutrophils 10.6, and CRP 83.1. Tissue necrosis is often associated with primary hepatic ASC, the clinical features of which may prompt an inappropriate diagnosis of hepatic abscess. The diagnostic uncertainty of a primary hepatic ASC with tissue necrosis versus a liver abscess is further exacerbated due to similarities on imaging modalities and in biochemical investigations [12].

In a case series by Zhang XF et al. documenting primary SCC of the liver, 19 patients underwent surgical intervention while 13 patients underwent palliative management [17]. Of those who underwent surgical intervention, 17 cases were freely accessible and only 2 papers commented on lymph node resection and histological evaluation [18,19]. In our case, had lymph node resection not occurred, glandular differentiation, and expression of CK 19 apparent in the lymph nodes would not have been identified. As such, had a right hemi hepatectomy with lymphadenectomy not been undertaken, this case would have been assigned to an inappropriate nomenclature, i.e., that of primary keratinizing SCC. With the benefit of hindsight, primary SCC of the liver may in fact be an even rarer entity.

Due to the aggressive nature of disease, individuals with ICC that demonstrate a significant squamous cell component have a poor prognosis [15]. The 1-year survival rate of primary intrahepatic adenosquamous cholangiocarcinoma is 18.8 % with <1 % surviving after the second year [12]. In contrast the 1-year, 2-year and 3-year survival rates of intrahepatic cholangiocarcinoma composed of predominantly glandular tissue is 32.8 %, 14.8 % and 9.5 % respectively [12]. Invasion into the liver parenchyma, early distant, or lymph node metastases may account for this [20]. Like other forms of cholangiocarcinoma, resection of the primary lesion with the potential for adjuvant treatment is indicated for ICC [15].

## 4 Conclusion

This article highlights the diagnostic challenges in determining newly classified rare primary liver malignancies, and their poor prognosis.

## Patient consent

Written informed consent was obtained from the patient for publication of this case report and accompanying images.

## Ethical approval

The study is exempt from ethical approval at out institution.

## Funding

N/A.

## Author contribution

Fionn Woulfe, Michael Devine, Adrian O'Sullivan all conceptualized the article and partook in writing and revising the manuscript. Rory Cortty and Brian Hayes analyzed the histology, identified appropriate specimens requested by the journal, and partook in the writing and revising of the manuscript.

## Guarantor

All five authors act as guarantor, independently and collectively for the content of this work.

## Research registration number

N/A.

## Conflict of interest statement

The authors have no conflicts of interest to declare.
